# Validity and Reliability of a Short Diet Questionnaire to Estimate Dietary Intake in Older Adults in a Subsample of the Canadian Longitudinal Study on Aging

**DOI:** 10.3390/nu10101522

**Published:** 2018-10-17

**Authors:** Anne Gilsing, Alexandra J. Mayhew, Hélène Payette, Bryna Shatenstein, Sharon I. Kirkpatrick, Krystle Amog, Christina Wolfson, Susan Kirkland, Lauren E. Griffith, Parminder Raina

**Affiliations:** 1 Department of Health Research Methods, Evidence, and Impact, McMaster University, Hamilton, ON L8S 4K1, Canada; anne.gilsing@maastrichtuniversity.nl (A.G.); mayhewaj@mcmaster.ca (A.J.M.); krystle.amog@gmail.com (K.A.); griffith@mcmaster.ca (L.E.G.); 2 McMaster Institute for Research on Aging, Hamilton, ON L8S 4L8, Canada; 3 Labarge Centre for Mobility in Aging, Hamilton, ON L8S 4K1, Canada; 4 Centre de Recherche sur le Vieillissement, CIUSSS de l’Estrie-CHUS Sherbrooke, Sherbrooke, QC J1J 3H5, Canada; Helene.Payette@USherbrooke.ca; 5 Département des Sciences de la Santé Communautaire, Faculté de Médecine et des; Sciences de la Santé, Université de Sherbrooke, Sherbrooke, QC J1H 5N4, Canada; 6 Centre de Recherche, Institut Universitaire de Gériatrie de Montréal, CIUSSS du Centre-Sud-de-l’Île-de-Montréal, Montréal, QC H3W 1W5, Canada; bryna.shatenstein@umontreal.ca; 7 Département de Nutrition, Université de Montréal, Montréal, QC H3T 1A8, Canada; 8 School of Public Health and Health Systems, University of Waterloo, Waterloo, ON N2L 3G1, Canada; sharon.kirkpatrick@uwaterloo.ca; 9 Department of Epidemiology and Biostatistics and Occupational Health, McGill University, Montreal, QC H3A 1A2, Canada; christina.wolfson@mcgill.ca; 10 Department of Community Health and Epidemiology, Dalhousie University, Halifax, NS B3H 4R2, Canada; susan.kirkland@dal.ca

**Keywords:** dietary assessment, 24 h recall, validity, reliability

## Abstract

This study assessed test-retest reliability and relative validity of the Short Diet Questionnaire (SDQ) and usability of an online 24 h recall among 232 participants (62 years ± 9.1; 49.6% female) from the Canadian Longitudinal Study on Aging (CLSA). Participants were asked to complete four 24 h dietary recalls (24HRs) using the Automated Self-Administered 24-h Dietary Assessment Tool (ASA24-Canada-2014), two SDQ administrations (prior to recalls one and four), and the System Usability Scale (SUS) for ASA24. For the SDQ administrations, Intraclass Correlation Coefficients ranged from 0.49 to 0.57 for nutrients and 0.35 to 0.72 for food groups. Mean intakes estimated from the SDQ were lower compared than those from the 24HRs. For nutrients, correlation coefficients were highest for fiber, calcium, and vitamin D (45–64 years: 0.59, 0.50, 0.51; >65 years: 0.29, 0.38, 0.49, *p* < 0.01); Kappas ranged from 0.14 to 0.37 in those 45–64 years and 0.17 to 0.32 in participants >65 years. Among the 70% who completed all recalls independently, the SUS indicated poor usability, though the majority reported feeling confident using ASA24. Overall, the SDQ captures intake with varying test-retest reliability and accuracy by nutrient and age. Further research is needed to inform use of a more comprehensive dietary measure in the CLSA.

## 1. Introduction

Although there is great scientific interest in the role of diet and nutrition in chronic disease prevention and healthy aging [1,2,3], dietary intake is arguably one of the most difficult exposures to adequately measure in population-based studies [4]. In addition to challenges associated with measuring diet in large studies regardless of the specific population [4], accurate dietary reporting in older adults can be further complicated by age-related declines in memory, cognitive capacity, and hearing and/or vision [5,6,7,8,9,10].

Age-related considerations guided the decision to implement a short dietary assessment tool to provide a rapid estimate of selected aspects of diet among participants in the Canadian Longitudinal Study on Aging (CLSA), one of the largest studies of older adults in the world. Launched in 2010, the CLSA is a national study that includes more than 50,000 community-dwelling men and women aged 45 to 85 who will be followed for at least 20 years, and undergo repeated waves of data collection every three years [11]. To balance data collection requirements, time constraints, and participant burden, the CLSA team developed the Short Diet Questionnaire (SDQ), a 36-item non-quantitative food frequency questionnaire focused on foods and nutrients of concern in adult populations, including total fat and fatty acid classes, dietary fiber, calcium, vitamin D, and fruits and vegetables [12].

The SDQ was developed as a brief dietary assessment instrument to avoid adding unnecessary participant burden to an already time-intensive data collection process. As a consequence, the SDQ is limited in detail, and like most brief diet questionnaires, does not query portion size, food preparation methods, or contextual information such as when meals are eaten. Though beneficial for reducing participant burden, short dietary assessment tools tend to capture diet with less accuracy than more comprehensive tools, necessitating evaluation to guide appropriate data analyses and interpretation. Estimates from the SDQ were previously evaluated against the mean of three non-consecutive 24 h dietary recalls (24HRs) in a subgroup of participants in the Québec Longitudinal Study on Nutrition and Successful Aging study (NuAge), aged 67 to 83 years [12]. Like all dietary screeners, the results suggest that the SDQ was a reasonably accurate, rapid approach for ranking usual frequencies of selected nutrients and foods (correlations ranged from *r* = 0.17 (*n*-3 fatty acids) to *r* = 0.45 (fruits and vegetables) [12]. A food frequency questionnaire should be tested for validity and reproducibility in each new target population and setting [4,13]. Given the broader age range of CLSA participants as compared to the NuAge sample as well as regional dietary variations, an updated study of the measurement properties (i.e., test-retest reliability and validity) of the SDQ was warranted to aid in interpretation of data from the CLSA.

Complementing the SDQ with more detailed dietary data may allow investigators to address additional lines of research, which is particularly relevant in the context of a rich data collection platform such as CLSA. While the collection of 24HRs has traditionally been cost-prohibitive in large studies due to the high interviewer burden [14], an internet based Automated Self-Administered 24-h Dietary Assessment Tool (ASA24) [15] has been developed. The ASA24 is a public-access, freely available tool that uses a computerized process to collect dietary recalls with no interviewer involvement, with the goal of enabling the collection of multiple 24HRs in large samples at low cost. A Canadian version of ASA24 that reflects the Canadian food supply was made available in 2014. The US version of ASA24 has been validated relative to true intake and also found to be feasible among adults [16,17,18]. Only one study examined the feasibility of the ASA24 in a sample of older adults (using a beta-version), and reported substantial challenges related to internet access and technology literacy. More research is needed to assess the usability of current versions of automated dietary assessment tools among older populations [19].

The aim of this study, the CLSA Diet Study, was thus to assess the test-retest reliability of the SDQ and the relative validity of the SDQ for selected nutrients (total fat and fatty acid classes, dietary fiber, calcium, and vitamin D) and fruits and vegetables compared to 24HRs collected using ASA24-Canada-2014 in a subsample of the CLSA cohort. In addition, the feasibility of using ASA24-Canada-2014 as an alternative or complementary dietary data collection method for future CLSA data collection waves was evaluated.

## 2. Methods

### 2.1. Study Sample

All CLSA participants provide a common set of information concerning many aspects of health and aging. A subset of 30,000 participants, belonging to the ”Comprehensive Cohort”, undergo additional in-depth examinations during an in-home interview followed by a visit to one of 11 data collection sites (DCS) where additional physical and psychological measurements are taken and, if consent is provided, biological specimens (i.e., blood and urine) are collected. Baseline data collection for the entire CLSA comprehensive cohort was conducted over three years and was completed in June 2015. The first follow-up measurement commenced in July 2015.

The CLSA Diet Study was conducted in a subsample of the Comprehensive cohort participants to make optimum use of existing data, infrastructure, and resources. Recruitment into the CLSA Diet Study took place between January and July 2016, during follow-up 1. During the in-home interview, participants of the McMaster University DCS in Hamilton, Ontario, were given an information brochure about the Diet Study. Approximately four weeks later, potential participants were provided with additional information about the study during their in-person visit to the McMaster University DCS; those who expressed interest were recruited into the study after providing informed consent. Participants without self-reported high-speed Internet access and those who reported to feel uncomfortable using computers were excluded. To meet participant enrolment targets (detailed below), a subset of younger individuals (48–67 years) who completed their CLSA follow-up measurement prior to January 2016 was recruited over the phone. No other selection criteria were imposed.

Sample size was based on the expected correlation between estimates from the SDQ and the 24HR. The minimum sample size for a desired lower limit of one-sided 95% confidence interval at *r* = 0.3 and a population correlation of 0.50 was calculated to be *n* = 57 per stratum [20]. Strata for age (45–64, and 65–87 + years of age) and sex (men, women) were considered. To account for the possibility of increased variability in the sample set and allowing for a potential 15% dropout rate, 265 participants were recruited into the study. Of these, 30 subjects dropped out during the study, and three subjects provided incomplete/unusable 24HR data, leaving a final overall sample size of 232. This study was approved by the Hamilton Integrated Research Ethics Board (project number 0750).

### 2.2. Data Collection

Participants were asked to complete four online non-consecutive 24HRs, using ASA24-Canada-2014, at one month intervals. We selected a total of four recalls based on recommendations for long term epidemiology studies that four to six dietary recalls over a year maximize precision [21], though the time period in our study was condensed. In addition to the SDQ collected as part of regular CLSA data collection prior to the start of this pilot study (SDQ1), participants also completed a second SDQ (SDQ2) prior to the final ASA24 recall.

### 2.3. SDQ

The development, pre-testing, and initial evaluation of the SDQ took place in a subsample of the NuAge Study and have been described elsewhere [12]. The SDQ contains 30 food items and six beverage items. It queries usual consumption frequency in the previous 12 months of food sources of fats, regular, and low-fat food choices, fiber, calcium, vitamin D, whole grains, calcium-fortified foods and beverages, and of a series of fruits and vegetables (see https://www.clsa-elcv.ca/researchers/data-collection for a copy of the questionnaire). Responses were given as a rate, i.e., number of times per time unit—day, week, month. No questions about portion-size were asked. Subjects completed an SDQ during their in-home interview as part of CLSA follow-up wave 1 (SDQ1), a few days to weeks prior to recruitment into the Diet Study.

The subset of younger participants (48–67 years) recruited over the phone had completed the in-home interview several weeks to months prior to enrollment in the Diet Study. To maintain comparable timing, these participants completed an additional SDQ, which served as SDQ1 for the purposes of this study, at the time of recruitment. All participants were contacted by phone to administer a second SDQ (SDQ2) before completing the final ASA24. The data from SDQ1 were cleaned by the diet study team and not by the CLSA-Statistical Analysis Centre because of its early release for the current study.

### 2.4. 24HRs

The ASA24-Canada-2014 was used to collect 24HRs [15]. The ASA24 was developed by the US National Cancer Institute and an adaptation to reflect the Canadian food supply was made available as of spring 2014. Modifications included adding foods and beverages unique to Canada and removing those not available in this country, changes to reflect differences in brand names in Canada compared to the U.S., and the addition of metric units. The version available at the time that data collection was conducted was ASA24-Canada-2014.

Using ASA24-Canada-2014, participants were prompted to recall the previous day’s intake from midnight to midnight. Using an adaptation of the US Department of Agriculture’s Automated Multiple-Pass Method (AMPM), ASA24 leads users through a series of passes to enhance accuracy and prompt the individual to remember all foods and beverages consumed. Multiple probes are used to collect details such as preparation method, allowing assignment of the closest match for foods eaten [15]. Participants choose portion sizes from images of foods and beverages in a variety of sizes.

Participants recruited in person completed the first ASA24-Canada-2014 recall at the end of their regular DCS visit in the presence of study staff who could guide participants on the process of completing a recall, answer questions, and provide assistance. The remaining recalls for these participants and all recalls for those recruited by phone were completed at home. Once a month for a total of three months, at unannounced time points, participants received an email request to log onto ASA24-Canada-2014 website and complete a recall for the previous day. An automated phone call was made in the morning to notify participants to check their email. If the participant was unable to complete the recall on the assigned day, up to five additional unannounced attempts were made. Participants could contact the Diet Study team for assistance by phone or email as needed. If participants were not able to complete ASA24-Canada-2014 independently, they were given the option to complete it with an interviewer over the phone.

### 2.5. ASA24 Feasibility Questionnaire

The usability of ASA24-Canada-2014 was assessed using items from the System Usability Scale (SUS) [22]. Administration of SUS was limited to participants who completed all recalls independently at home to obtain a measure of the feasibility of self-administration. SUS is a 10-item questionnaire with five response options ranging from strongly agree to strongly disagree. Questions cover a variety of aspects of usability, including the need for support, training, and complexity. As per the SUS manual, an overall score was calculated ranging from 0–100. The SUS does not provide insights into specific usability challenges, but does provide an overall indicator of usability. Based on a review of existing usability studies, normative data have been produced to allow SUS ratings to be positioned relative to other systems. Systems that are at least passable have SUS scores above 70.

### 2.6. Nutrient Calculations

Nutrient intake was calculated from the SDQ as described by [12] using participants’ reported frequencies of consumption of each SDQ item, for standard (medium) portion sizes estimated from a full food frequency questionnaire (FFQ) [23] administered in the NuAge study [24], and a nutrient database based on the 2007b Canadian Nutrient File. Nutrient calculation was restricted to the nutrients targeted by the SDQ (calcium, vitamin D, dietary fiber, total fat, cholesterol, and mono- and poly-unsaturated fatty acids).

Nutrient intakes from the 24HR data were calculated using the mean of the four administrations, and based on the 2015 version of CNF. Following cleaning procedures recommended by the National Cancer Institute [25] based on processes used for the National Health and Nutrition Examination Survey, 20 subjects were removed from the relative validity analyses (final sample *n* = 200).

### 2.7. Statistical Analyses

Frequencies and percentages as well as means and standard deviations (SD) were used to describe categorical and continuous variables, respectively.

For the test-retest reliability of the SDQ, an intra-class correlation coefficient (ICC) was calculated for nutrients targeted by the SDQ. These included dietary fiber, calcium, vitamin D, total fat, cholesterol, mono- and poly-unsaturated fatty acids and saturated fatty acids). Similar statistics were calculated for (combinations of) SDQ line items. Line items were grouped together into food categories. The ICC tested the level of agreement between nutrient estimates from SDQ1 and SDQ2. A weighted kappa was calculated to assess the agreement between age and sex specific quartiles of nutrient consumption. All reliability analyses were stratified by sex (male, female) and age (45–64 years, 65–87 years).

To assess relative validity, daily nutrient intake estimates derived from the SDQ2 were compared to the mean of four 24HRs using Spearman Rank correlations. Nutrient intakes from the SDQ and the mean of 24HRs were divided into quartiles, and the degree of agreement was evaluated using weighted Kappa. Analyses were stratified by age (45–64; 65–87 years) and sex.

Linear weights were used for the weighted kappa coefficients and 2-way mixed effects models with absolute agreement were used for the ICC calculations. The ICC and weighted kappa were interpreted following the guidelines from Landis and Koch [26] (0.01 to 0.2, slight agreement; 0.21 to 0.40, fair agreement; 0.41 to 0.60, moderate agreement; 0.61 to 0.80, substantial agreement; 0.81 to 1.0, almost perfect or perfect agreement). Statistical analysis was performed using Statistical Package for Social Sciences (SPSS) software (version 24, SPSS, Inc., Chicago, IL, USA).

Multiple imputation was used to impute missing values for both time points using thee SPSS multiple imputation function. Five imputations were calculated using linear regression. Variables included in the regression model were age group, sex, and the number of times per day each of the 36 SDQ items were consumed. Multiple imputation was performed separately for SDQ1 and SDQ2.

## 3. Results

Table 1 displays the sociodemographic characteristics of the 232 study participants included in the reliability analyses. We recruited equal numbers of males and females, but due to the higher drop-out rates among younger adults, 55.6% of our sample was 65–87 years. More than three quarters of participants had completed post-secondary education, almost half reported a household income greater than $100,000 CAD, and 78% were married or living in common law relationships. The majority were classified as overweight or obese based on their body mass index (48.3% and 25.9%, respectively), 9% were current smokers and 80% reported consuming alcohol on a regular basis. Of the 232 subjects included in the analyses, 29 had missing data for one or several items for SDQ1 and 17 had missing data for SDQ2.

### 3.1. Test-Retest Reliability

The ICC of derived nutrients between SDQ1 and SDQ2 ranged from 0.49 (95% confidence interval (CI) 0.38 to 0.59) for mono unsaturated fatty acids (MUFA) to 0.60 (95% CI 0.51 to 0.68) for cholesterol across all participants (Table 2). There were no consistent patterns of higher or lower correlations for younger (ICC range of 0.44 (95% CI 0.26 to 0.59) for MUFA, to 0.62 (95% CI 0.48 to 0.73) for cholesterol) versus older adults (ICC range of 0.51 (95% CI 0.37 to 0.63) for PUFA, 0.60 (95% CI 0.46 to 0.70) for Vitamin D). The weighted kappa values for the full sample were between 0.35 (95% CI 0.27 to 0.44) for PUFA and 0.44 (95% CI 0.36 to 0.52) for cholesterol (Table 2). No notable differences were observed between men and women [27].

For the combined SDQ line item groups, one food group (total fresh and processed meat and fish) had fair test-retest reliability (ICC of 0.35 (95% CI 0.23 to 0.46). Six food groups had moderate test-retest reliability (ICC between 0.40 to 0.59): fresh red meat; total fresh meat and fish; cheese; vegetables excluding potatoes; fruit juice; and calcium supplemented foods and drinks. The remaining five food groups had substantial test-retest reliability (ICC between 0.60 and 0.75): milk; eggs; yoghurt; sweet desserts and chocolate; and sweet and salty snacks and desserts (Table 3). When comparing males versus females and younger versus older adults, there was no clear evidence of differing test-retest reliability [27].

A wide range of ICC values were estimated for the agreement between SDQ1 and SDQ2 for individual SDQ line items. Six items had fair test-retest reliability (ICC < 0.40), 17 items had moderate test re-test reliability (ICC between 0.40 and 0.59), and 13 items had higher substantial test-retest reliability (ICC between 0.60 and 0.75) (Supplementary Table S1).

### 3.2. Validity

The mean intakes of selected nutrients as measured by SDQ2 and 24HRs, stratified by age (45–64, 65–87 years), are shown in Table 4. In both age groups, intakes estimated from the SDQ were significantly lower compared to the 24HRs for all nutrients. Spearman correlations between the SDQ and 24HRs were higher in those aged 45–64 years compared to those 65–87 years, except for monounsaturated fat and saturated fat. Dietary fiber, calcium, and vitamin D showed the strongest correlations in both age groups (45–64 year: *r* = 0.59, 0.50, 0.51, respectively; 65–87 years: *r* = 0.29, 0.38, 0.49, respectively). There were no sex differences, except for vitamin D, which showed a stronger correlation in men (0.64) compared to women (0.31) [27]. Age differences were smaller for the weighted kappa statistics, with the exception of dietary fiber and calcium, but there were no notable differences observed between men and women [27]. Kappas ranged from 0.14–0.37 in those 45–64 years and from 0.17–0.32 in participants 65–87 years, indicating slight to fair agreement.

### 3.3. Feasibility of the ASA24

A total of 220 of 232 participants completed four 24HRs, of whom 79 (36%) completed one or more recalls over the phone. Of the 123 who completed all four recalls independently at home and responded to the usability questionnaire, 111 completed the SUS scale, with a mean SUS score of 56.8 (SD 12.4) [27]. The results from the individual SUS items are depicted in Table 5. Almost half (45.1%) of respondents indicated that they would not use ASA24 frequently, 50.4% found ASA24 unnecessarily complex, and 43.4% reported that ASA24 was cumbersome to use. The majority of participants felt confident using the website (64.3%), and did not feel they needed technical support (77%).

## 4. Discussion

Our results suggest that among older adults, the SDQ captures intake with varying reliability and accuracy by dietary component and age group. The results were most promising for dietary fiber, vitamin D, and calcium, especially for those under 65 years. Further research is needed to inform the implementation of comprehensive technology-enabled dietary assessment tools among this population.

The SDQ assesses the frequency of consumption of key foods and nutrients that have been the focus of nutritional health promotion activities in middle aged and older adults. The test-retest reliability of the SDQ is dependent on the operationalization of the dietary variables, as evident in the differences observed for food groups versus nutrients. Our findings suggest that reliably assessing usual intake of individual line items is more challenging than estimating intake of nutrients and food items and groupings. This is in line with previous research showing lower reliability values for intake estimates of individual line items compared to nutrients [4,28,29]. Careful consideration should be given when using intake data from individual line items in the CLSA or similar populations, as for some items, there was little agreement between SDQ administrations. These items were rarely eaten (e.g., pâtés, cretons and terrines, calcium-fortified juices, calcium-fortified milk, and whole milk) or the question was worded vaguely (e.g., “other vegetables”), which could have made it difficult for participants to provide accurate responses.

No consistent differences in test-retest reliability were found between younger versus older adults, or males versus females, suggesting that these subgroups have a similar ability to respond consistently to the SDQ over time. Differences between males and females and different age groups in test-retest reliability of short diet screeners and full FFQs are not commonly tested. However, a previous study reported that estimates of reproducibility of FFQs is similar between males and females [30]. Since the test-retest results reflect both the performance of the SDQ and actual changes in dietary intake over time, the observed test-retest reliability values are likely to be conservative estimates.

The SDQ showed good relative validity compared to the 24HR data for ranking participants aged 45 to 64 according to their intake of vitamin D, calcium, and fiber. Correlation coefficients for relative validity of diet questionnaires should ideally be in the range of 0.5–0.7 [4]. The correlation coefficients for these nutrients were lower in participants aged 65–87 years and were considered to have moderate validity. It must be kept in mind however, that correlations are measures of association and that both true intake and error could be correlated between the SDQ and 24HRs, inflating observed measures of association. For this reason, it is suggested that correlations not be relied upon as the sole source of information on relative validity [31]. We also examined Kappa statistics for the association between the mean of four dietary recalls and the ASA, showing similar results.

Positioning these findings in the context of previous research is limited by the heterogeneity of short dietary assessment tools, including their purposes and target populations, reference time frames, the inclusion of portion sizes, and the design of the validation studies [32]. Although short diet questionnaires have been shown to have greater underestimation of nutrient intake compared to full length FFQs, many have been shown to adequately rank subjects along the distribution of intakes. The lower correlation coefficients in those over 65 years observed in this study were in the same order of magnitude with those from a previous validation study of the SDQ in a subset of the NuAge study aged 67–84 years [12]. Though there have been a limited number of studies that have assessed the performance of other short FFQs in people over 65 years, most found moderate validity for nutrients and food groups of interest [7,8,33]. However, it is unclear whether the lower correlations in older versus younger participants are due to challenges in completing the SDQ, 24HRs, or both. It has been suggested that older adults find 24HRs more difficult to complete compared to FFQs, as they may have more issues with short-term compared to long-term recalls [4,6,9,10]. Using an internet-based automated self-administered 24HR could have also added another level of complexity. Nearly half of the participants older than 65 years completed one or more recalls over the phone (44%), which anecdotally was often due to limited computer literacy. The median age of the phone participants was higher than that of the overall group of senior participants (76 versus 68 years, respectively). Lower correlation coefficients between the SDQ and the recall data were observed among phone participants for almost all nutrients compared to older adults who completed the recalls independently, suggesting an age effect in recalling or reporting short-term diet.

New analytical procedures have recently been developed to increase the usability of screener-type questionnaires by calibrating them to data collected using a more comprehensive, and presumably more accurate, assessment method [34,35]. We explored whether the data from the 24HRs collected in this study could be used to generate scoring algorithms to re-scale data from the SDQ using methods described previously [34,35]. The performance of the algorithms was tested in a subset of the NuAge cohort [12], but our results did not suggest notable improvements of mean nutrient intake estimates or relative validity of the estimates over the direct estimation of nutrients after linkage to the CNF. Given the desire for short tools in many studies, further efforts to identify strategies to improve the data they capture seems warranted.

Alternatively, technology-enabled advancements may make it possible to collect more detailed dietary data in large studies like the CLSA. However, while the availability of ASA24-Canada-2014 overcomes key limitations that have hampered the use of interviewer-administered recalls (including the need for trained interviewers and coders) in large-scale research, its use in older adults may introduce new challenges related to cognitive abilities and computer literacy. Participants who completed all ASA24 recalls in this study independently rated the usability of the 2014 version of ASA24-Canada as poor (mean SUS score of 57). Over 30% of participants completed one or more of their recalls over the phone either because of technological issues, computer literacy issues, or because the recalls took too long to complete independently. Of these participants, 6.3% were women younger than 65 years, 45% were women 65 years and older, 22% were men below the age of 65, and 26% were men 65 years and older. Informal feedback from participants highlighted issues with the user-friendliness of the system, technological barriers to completing recalls, and limitations of food selection options. A previous study using the Beta version of ASA24 in older adults (55 to 80 years) also found technology literacy to be a barrier for implementation and half of the participants preferred a traditional 24HR over ASA24 [19]. Since completion of the CLSA Diet Study, an updated version of ASA24-Canada was released in Fall 2016 including a French version and an improved interface that overcomes some of the technological issues in this study. Specifically, the new version no longer requires the Microsoft Silverlight plugin (support for which was withdrawn by the popular Internet browsers in recent years) that prevented numerous participants from accessing the website on their personal computer. The new version is available in both English and French and can be completed on laptops and desktop computers, as well as mobile devices. Though improvements in usability are expected based on the updates, ASA24-Canada-2016 has not yet been tested in older adults. Lessons learned in this and other studies of ASA24 in varied Canadian populations and recommendations to inform future research and usage of such tools have been published elsewhere [36]. Further testing is needed to better understand the particular strategies to be used in populations of older adults.

The findings of this study should be interpreted in light of several considerations. The study made use of a relatively large sample, but participants were already committed to the CLSA, potentially increasing their willingness to complete the dietary assessment tools. Intakes estimated from the SDQ were compared to the mean of four 24HRs. Such comparative validation is imperfect, as no self-report instrument represents true intake and errors in the two instruments that depend on memory are likely to be correlated. However, it is difficult to improve upon this because the nutrients and food groups of interest do not have recovery biomarkers providing unbiased indications of true intake, and other methods of obtaining estimates of true intake (e.g., observation) are not feasible in studies such as this. Additionally, the foods used for creating line items in the original FFQ and hence the SDQ were based on population food consumption data from the Quebec nutrition survey conducted in the 1990s and analysed using the 2007b CNF [12], whereas ASA24 was linked to 2015 version of the CNF for nutrient analysis. Changes in food consumption patterns and food composition over time could have attenuated the validity estimates reported.

Our results suggest that among older adults, the SDQ captures intake with varying reliability and accuracy by dietary component and age group. The results were most promising for dietary fiber, vitamin D, and calcium, especially for those under 65 years. While the SDQ is a cost-effective tool with low participant burden, its potential applications in large population-based studies are limited due to its narrow scope and inability to describe overall population dietary intake. In population-based studies, the SDQ could serve as a tool for rapid assessment of usual consumption frequencies of the foods and food groups included in the questionnaire. It could also serve to compare changes in patterns of consumption over time. As the population ages, the validity of the SDQ in the oldest age groups should continue to be monitored.

Indeed, diet assessment in older adults is a challenge; various factors relating to age should be considered and the choice of instruments should balance participant burden with the quality of dietary assessment. The emergence of innovative technology-enabled tools offers opportunities but also poses operational challenges in populations of older adults; further research is needed to examine how to optimize the use of such tools for capturing detailed dietary intake data in older adults. This is increasingly important as diet-related chronic diseases continue to contribute greatly to morbidity and mortality in Canada and elsewhere.

## Figures and Tables

**Table 1:** Socio-demographic characteristics of the Canadian Longitudinal Study on Aging (CLSA) Diet Study participants.

	*n* (Total = 232)	%
**Sex**		
Males	115	49.6
Females	117	50.4
**Age**		
5–64 years	103	44.4
65 + years	129	55.6
**Education**		
Less than secondary	6	2.6
Secondary	22	9.5
Some post-secondary	23	10.0
Post-secondary	180	77.9
**Household Income**		
Less than $20,000	3	1.4
$20,000–$49,999	35	16.0
$50,000–$99,999	77	35.2
$100,000–$149,999	59	26.9
Greater than $150,000	45	20.5
**Marital Status**		
Single, never married, or never lived with partner	11	5.0
Married, living common law	172	78.5
Widowed, divorced, separated	36	16.4
**Body Mass Index**		
Underweight (<18.5 kg/m^2^)	2	0.9
Normal weight (18.5–24.9 kg/m^2^)	58	25.0
Overweight (25.0–29.9 kg/m^2^)	112	48.3
Obese (>30.0 kg/m^2^)	60	25.9
**Smoking Status**		
Current smoker	16	9.1
Former smoker	98	56.0
Never smoker	61	34.9
**Alcohol Consumption in Past 12 Months**		
Regular drinker (at least once a month)	176	80.4
Occasional drinker	24	11.0
Did not drink in the past 12 months	19	8.7

**Table 2:** Estimated intakes of nutrients of interest from SDQ1 and SDQ2 and test-retest reliability between administrations, CLSA Diet Study (*n* = 232).

	SDQ1		SDQ2		ICC (95% CI)	Weighted Kappa (95% CI)
	Mean	SD	Mean	SD		
**Middle Aged (45–64 years; *n* = 103)**						
Dietary fiber (g)	14.3	4.8	13.2	5.1	0.57 (0.42–0.69)	0.41 (0.30–0.52)
Protein (g)	65.4	20.3	63.5	16.6	0.49 (0.32–0.63)	0.33 (0.20–0.46)
Calcium (mg)	740.4	381.6	750.0	317.8	0.53 (0.37–0.66)	0.43 (0.31–0.55)
Fat (g)	53.5	17.3	51.2	14.4	0.49 (0.32–0.63)	0.36 (0.23–0.49)
Vitamin D (ug)	4.7	3.0	5.0	2.5	0.54 (0.38–0.67)	0.40 (0.28–0.52)
Cholesterol (mg)	221.4	84.8	208.9	74.4	0.62 (0.48–0.73)	0.45 (0.33–0.58)
MUFA (mg)	19.8	6.9	18.8	5.7	0.44 (0.26–0.59)	0.34 (0.21–0.47)
PUFA (mg)	10.3	3.5	10.0	3.3	0.52 (0.36–0.66)	0.43 (0.31–0.54)
Saturated fat (mg)	19.2	6.6	18.1	5.6	0.57 (0.41–0.70)	0.47 (0.42–0.52)
**Seniors (65+ years; *n* = 129)**						
Dietary fiber (g)	15.5	5.0	15.2	5.1	0.55 (0.41–0.66)	0.34 (0.23–0.45)
Protein (g)	65.7	17.2	67.0	17.4	0.57 (0.44–0.68)	0.38 (0.27–0.48)
Calcium (mg)	758.6	315.7	845.4	361.0	0.57 (0.42–0.68)	0.37 (0.25–0.49)
Fat (g)	53.2	16.4	54.6	17.8	0.53 (0.39–0.65)	0.36 (0.26–0.47)
Vitamin D (ug)	5.1	2.6	5.8	2.7	0.60 (0.46–0.70)	0.40 (0.29–0.51)
Cholesterol (mg)	213.6	74.0	220.8	81.7	0.59 (0.46–0.70)	0.42 (0.31–0.53)
MUFA (mg)	19.6	6.5	19.8	7.1	0.53 (0.39–0.65)	0.38 (0.27–0.48)
PUFA (mg)	10.4	3.6	10.4	3.4	0.51 (0.37–0.63)	0.28 (0.16–0.40)
Saturated fat (mg)	17.3	6.0	19.3	7.0	0.54 (0.38–0.66)	0.35 (0.29–0.40)

SDQ1, Short Diet Questionnaire 1; SDQ2, Short Diet Questionnaire 2; MUFA, monounsaturated fatty acids; PUFA, polyunsaturated fatty acids.

**Table 3:** Estimated intakes of line items and combinations of line items from SDQ1 and SDQ2 and test-retest reliability between administrations, CLSA Diet Study (*n* = 232).

	SDQ1		SDQ2		ICC (95%) CI
SDQ Line Item Combination	Mean	SD	Mean	SD	
Fresh red meat ^1^	0.30	0.27	0.30	0.22	0.47 (0.36–0.56)
Total fresh meat and fish ^2^	0.62	0.34	0.62	0.30	0.58 (0.48–0.66)
Total fresh and processed meat, and fish ^3^	0.78	0.46	0.77	0.43	0.35 (0.23–0.46)
Milk ^4^	0.90	0.97	1.05	0.85	0.62 (0.54–0.70)
Cheese ^5^	0.52	0.35	0.52	0.45	0.42 (0.31–0.52)
Eggs ^6^	0.32	0.24	0.32	0.24	0.70 (0.63–0.76)
Yoghurt ^7^	0.52	0.46	0.47	0.42	0.72 (0.65–0.77)
Vegetables excluding potatoes ^8^	1.89	1.07	1.82	1.00	0.49 (0.39–0.58)
Fruit juice ^9^	0.41	0.56	0.42	0.71	0.50 (0.40–0.59)
Calcium supplemented food ^10^	0.09	0.32	0.09	0.34	0.44 (0.33–0.54)
Sweet desserts and chocolate ^11^	0.41	0.39	0.39	0.42	0.60 (0.51–0.68)
Sweet and salty snacks and desserts ^12^	0.72	0.54	0.77	0.55	0.65 (0.57–0.72)

ICC, Intraclass Correlation Coeficient. ^1^ Beef, pork, and other meats (veal, lamb, and game). ^2^ Beef, pork, other meats (veal, lamb, and game), chicken, turkey, and fish. ^3^ Beef, pork, other meats (veal, lamb, and game), chicken, turkey, and fish, sauces, hot dogs, ham, smoker meat, bacon, pates, cretons, and terrines. ^4^ Calcium fortified milk, whole milk, and 2%, 1%, and skim milk. ^5^ Regular fat cheese and low-fat cheese. ^6^ Omega 3 enriched eggs and regular eggs. ^7^ Low fat yogurt and regular fat yogurt. ^8^ Green salad, carrots, and vegetables other than green salad, carrots, and potatoes. ^9^ Calcium enriched juice and 100% pure fruit juice. ^10^ Calcium fortified foods, calcium fortified juices, calcium fortified milk, and calcium fortified beverages. ^11^ Cakes, pies, doughnuts, pastries, cookies, muffins and chocolate bars. ^12^ Cakes, pies, doughnuts, pastries, cookies, muffins, chocolate bars, ice cream, ice milk, frozen yogurt, milk-based desserts (puddings), and salty snacks (regular chips, crackers).

**Table 4:** Association between nutrient estimates from the SDQ and the mean of four dietary recalls collected using the Automated Self-Administered 24-h Dietary Assessment Tool (ASA24-Canada-2014), CLSA Diet Study (*n* = 200).

	SDQ2		24-HRs		Spearman *R* *	Weighted Kappa
	Mean	SD	Mean	SD		
**Middle aged (45–64 years; *n* = 96)**						
Dietary fiber (g)	13.3	5.3	19.8	8.1	0.59	0.36
Calcium (mg)	734	312	899	353	0.50	0.37
Vitamin D (ug)	4.8	2.5	5.5	4.5	0.51	0.28
Total fat (g)	50.7	14	75.4	20	0.26	0.23
Cholesterol (mg)	210	76	273	105	0.31	0.20
Monounsaturated fat (g)	18.6	5.6	27.9	7.6	0.21	0.16
Polyunsaturated fat (g)	9.9	3.2	16.3	5.8	0.29	0.14
Saturated fat (g)	17.8	5.5	24.8	7.6	0.26	0.18
**Seniors (65+ years; *n* = 104)**						
Dietary fiber (g)	15.5	4.8	21.4	4.1	0.29	0.17
Calcium (mg)	849	352	922	351	0.38	0.22
Vitamin D (ug)	5.9	2.5	6.6	4.3	0.49	0.32
Total fat (g)	55.5	17	73.7	28	0.25	0.21
Cholesterol (mg)	221	82	268	112	0.24	0.17
Monounsaturated fat (g)	20.1	6.4	26.4	10.6	0.23	0.16
Polyunsaturated fat (g)	10.9	3.5	17.1	8.5	0.17	0.17
Saturated fat (g)	19.4	6.3	23.8	9.6	0.31	0.25

24-HRs: 24 h dietary recalls. * All correlations were statistically significant at *p* < 0.05.

**Table 5:** Individual scores from the System Usability Scale (SUS) to evaluate the usability of ASA24, CLSA Diet Study (*n* = 123).

SUS Item	Disagree (%)	Neutral (%)	Agree (%)
I think that I would like to use the diet survey frequently	45.1	37.2	17.7
I found the diet survey unnecessarily complex	42.0	17.0	50.4
I thought the diet survey was easy to use	31.9	17.7	50.4
I think I would need the support of a technical person to be able to use the diet survey	77.0	10.6	12.4
I found the various functions of the ASA24-Canada were well integrated.	17.7	31.0	51.3
I thought there was too much inconsistency in the ASA24-Canada	55.8	37.2	7.1
I imagine that most people would learn to use the ASA24-Canada very quickly	29.2	26.5	44.2
I found the ASA24-Canada very cumbersome to use	39.8	16.8	43.4
I felt very confident using the ASA24-Canada	12.5	23.2	64.3
I needed to learn a lot of things before I could use the ASA24-Canada	71.7	20.4	8.0

## Supplementary Materials

The following are available online at http://www.mdpi.com/2072-6643/10/10/1522/s1, Table S1: Estimated intake of individual line items from SDQ1 and SQ2 and test-retest reliability between administrations, CLSA Diet Study (*n* = 232).
