# Supplementary material for: Validity and Reliability of a Short Diet Questionnaire to Estimate Dietary Intake in Older Adults in a Subsample of the Canadian Longitudinal Study on Aging
Source: Nutrients. 2018 Oct 17;10(10):1522. doi: 10.3390/nu10101522 (PMC6213467; doi:10.3390/nu10101522)
Supplement: Supplementary file 1 [file nutrients-10-01522-s001.pdf]

Supplementary Table S1: Estimated intake of individual line items from SDQ1 and SQ2 and test-retest reliability between administrations, CLSA Diet Study (*n* = 232).

SDQ Line Item	SDQ1		SDQ2		ICC (95%) CI
	Mean	SD	Mean	SD	
High fiber breakfast cereals	0.45	0.41	0.49	0.43	0.70 (0.62–0.91)
Whole wheat breads, bran breads, multigrain breads	0.80	0.68	0.73	0.53	0.40 (0.29–0.89)
Beef, pork (ground, hamburgers, roast beef, steak)	0.28	0.26	0.28	0.22	0.45 (0.34–0.87)
Other meats (veal, lamb, game)	0.03	0.05	0.03	0.06	0.54 (0.44–0.89)
Chicken, turkey	0.32	0.19	0.32	0.19	0.70 (0.63–0.78)
Salmon, trout, sardines, herring, tuna, mackerel	0.19	0.17	0.18	0.17	0.62 (0.54–0.84)
Sausages, hot dogs, ham, smoked meat, bacon	0.14	0.17	0.13	0.13	0.51 (0.41–0.87)
Patés, cretons, terrines…	0.03	0.26	0.01	0.03	0.01 (-0.12–0.87)
Sauces and gravies (brown, white, BBQ)	0.10	0.13	0.13	0.16	0.40 (0.28–0.79)
Omega-3eggs	0.12	0.21	0.13	0.23	0.69 (0.62–0.90)
All egg dishes except omega 3 eggs (eggs, omelette)	0.21	0.22	0.20	0.21	0.70 (0.63–0.88)
Legumes (beans, peas, lentils)	0.33	0.26	0.33	0.28	0.46 (0.35–0.81)
Nuts, seeds and peanut butter	0.60	0.48	0.60	0.46	0.54 (0.44–0.84)
Fruit (fresh, frozen, canned)	1.46	0.98	1.36	0.95	0.56 (0.46–0.85)
Green salad	0.57	0.38	0.57	0.34	0.48 (0.37–0.88)
Potatoes (boiled, mashed or baked)	0.30	0.22	0.30	0.25	0.64 (0.56–0.93)
French fries or pan-fried potatoes, poutine	0.07	0.08	0.07	0.07	0.29 (0.17–0.83)
Carrots (fresh, frozen, canned)	0.36	0.28	0.32	0.29	0.69 (0.62–0.91)
Other vegetables (except carrots, potatoes or salad)	0.98	0.87	0.92	0.69	0.32 (0.20–0.85)
All low-fat cheeses	0.09	0.21	0.10	0.20	0.56 (0.46–0.89)
All regular cheeses	0.43	0.32	0.41	0.32	0.60 (0.51–0.90)
Yogurt (low-fat)	0.35	0.43	0.31	0.40	0.62 (0.53–0.85)
Yogurt (regular)	0.18	0.32	0.16	0.30	0.69 (0.62–0.88)
Calcium-fortified foods (soy pudding)	0.01	0.10	0.02	0.12	0.56 (0.46–0.85)
Milk-based desserts	0.12	0.17	0.15	0.19	0.66 (0.58–0.85)
Salty snacks (regular chips, crackers)	0.19	0.22	0.23	0.24	0.57 (0.47–0.82)
Cakes, pies, doughnuts, pastries, cookies, muffins…	0.31	0.33	0.28	0.32	0.57 (0.48–0.85)
Chocolate bars	0.10	0.17	0.11	0.22	0.55 (0.46–0.86)
Butter or regular margarine	0.70	0.64	0.69	0.68	0.46 (0.35–0.92)
Regular vinaigrettes, salad dressings, mayonnaise, dips	0.45	0.31	0.48	0.34	0.59 (0.50–0.86)
Calcium-fortified juices	0.05	0.18	0.07	0.23	0.15 (0.02–0.80)
100% pure fruit juices (orange, grapefruit or tomato)	0.38	0.50	0.33	0.46	0.68 (0.60–0.92)
Calcium-fortified milk (35% more calcium)	0.00	0.00	0.05	0.27	0.00 (-0.13–0.00)
Whole milk 3.25% m.f. for drinking	0.02	0.15	0.04	0.21	0.21 (0.08–0.90)
2%, 1%, skim milk for drinking	0.88	0.98	0.95	0.79	0.68 (0.60–0.90)
Other calcium-fortified beverages (soy drink)	0.08	0.31	0.08	0.33	0.46 (0.36–0.86)
